# Tissue Glucocorticoid Metabolism in Adrenal Insufficiency: A Prospective Study of Dual-release Hydrocortisone Therapy

**DOI:** 10.1210/clinem/dgad370

**Published:** 2023-06-20

**Authors:** Rosemary A Dineen, Julie Martin-Grace, Khalid Mohamed Saeed Ahmed, Angela E Taylor, Fozia Shaheen, Lina Schiffer, Lorna C Gilligan, Gareth G Lavery, Isolda Frizelle, Anjuli Gunness, Aoife Garrahy, Anne Marie Hannon, Paal Methlie, Sverre Husebye Eystein, Paul M Stewart, Jeremy W Tomlinson, James M Hawley, Brian G Keevil, Michael W O’Reilly, Diarmuid Smith, John McDermott, Marie-Louise Healy, Amar Agha, Agnieszka Pazderska, James Gibney, Lucy-Ann Behan, Chris J Thompson, Wiebke Arlt, Mark Sherlock

**Affiliations:** Academic Department of Endocrinology, Beaumont Hospital/Royal College of Surgeons in Ireland, Dublin, D09 YD60, Ireland; Academic Department of Endocrinology, Beaumont Hospital/Royal College of Surgeons in Ireland, Dublin, D09 YD60, Ireland; Robert Graves Institute of Endocrinology, Tallaght University Hospital, Dublin, D24 TP66, Ireland; Institute of Metabolism and Systems Research, University of Birmingham, Birmingham B15 2TT, UK; Institute of Metabolism and Systems Research, University of Birmingham, Birmingham B15 2TT, UK; Institute of Metabolism and Systems Research, University of Birmingham, Birmingham B15 2TT, UK; Institute of Metabolism and Systems Research, University of Birmingham, Birmingham B15 2TT, UK; Institute of Metabolism and Systems Research, University of Birmingham, Birmingham B15 2TT, UK; Robert Graves Institute of Endocrinology, Tallaght University Hospital, Dublin, D24 TP66, Ireland; Robert Graves Institute of Endocrinology, Tallaght University Hospital, Dublin, D24 TP66, Ireland; Academic Department of Endocrinology, Beaumont Hospital/Royal College of Surgeons in Ireland, Dublin, D09 YD60, Ireland; Academic Department of Endocrinology, Beaumont Hospital/Royal College of Surgeons in Ireland, Dublin, D09 YD60, Ireland; Department of Clinical Science, University of Bergen, 5021 Bergen, Norway; Department of Clinical Science, University of Bergen, 5021 Bergen, Norway; Faculty of Medicine and Health, University of Leeds, Leeds LS2 9JT, UK; Oxford Centre for Diabetes, Endocrinology and Metabolism, NIHR Oxford Biomedical Research Centre, Churchill Hospital, University of Oxford, Oxford OX3 7LE, UK; Department of Clinical Biochemistry, University Hospital of South Manchester, Manchester Academic Health Science Centre, The University of Manchester, Manchester M23 9LT, UK; Department of Clinical Biochemistry, University Hospital of South Manchester, Manchester Academic Health Science Centre, The University of Manchester, Manchester M23 9LT, UK; Academic Department of Endocrinology, Beaumont Hospital/Royal College of Surgeons in Ireland, Dublin, D09 YD60, Ireland; Academic Department of Endocrinology, Beaumont Hospital/Royal College of Surgeons in Ireland, Dublin, D09 YD60, Ireland; Department of Endocrinology, Connolly Hospital, Dublin, D15 X40D, Ireland; Department of Endocrinology, St James Hospital, Dublin, D08 K0Y5, Ireland; Academic Department of Endocrinology, Beaumont Hospital/Royal College of Surgeons in Ireland, Dublin, D09 YD60, Ireland; Department of Endocrinology, St James Hospital, Dublin, D08 K0Y5, Ireland; Robert Graves Institute of Endocrinology, Tallaght University Hospital, Dublin, D24 TP66, Ireland; Robert Graves Institute of Endocrinology, Tallaght University Hospital, Dublin, D24 TP66, Ireland; Academic Department of Endocrinology, Beaumont Hospital/Royal College of Surgeons in Ireland, Dublin, D09 YD60, Ireland; Institute of Metabolism and Systems Research, University of Birmingham, Birmingham B15 2TT, UK; Medical Research Council London, Institute of Medical Sciences, London W12 0NN, UK; Academic Department of Endocrinology, Beaumont Hospital/Royal College of Surgeons in Ireland, Dublin, D09 YD60, Ireland

**Keywords:** cortisol, adrenal insufficiency, 11beta-hydroxysteroid dehydrogenase, metabolism

## Abstract

**Background:**

Patients with adrenal insufficiency (AI) require life-long glucocorticoid (GC) replacement therapy. Within tissues, cortisol (F) availability is under the control of the isozymes of 11β-hydroxysteroid dehydrogenase (11β-HSD). We hypothesize that corticosteroid metabolism is altered in patients with AI because of the nonphysiological pattern of current immediate release hydrocortisone (IR-HC) replacement therapy. The use of a once-daily dual-release hydrocortisone (DR-HC) preparation, (Plenadren®), offers a more physiological cortisol profile and may alter corticosteroid metabolism in vivo.

**Study Design and Methods:**

Prospective crossover study assessing the impact of 12 weeks of DR-HC on systemic GC metabolism (urinary steroid metabolome profiling), cortisol activation in the liver (cortisone acetate challenge test), and subcutaneous adipose tissue (microdialysis, biopsy for gene expression analysis) in 51 patients with AI (primary and secondary) in comparison to IR-HC treatment and age- and BMI-matched controls.

**Results:**

Patients with AI receiving IR-HC had a higher median 24-hour urinary excretion of cortisol compared with healthy controls (72.1 µg/24 hours [IQR 43.6-124.2] vs 51.9 µg/24 hours [35.5-72.3], *P* = .02), with lower global activity of 11β-HSD2 and higher 5-alpha reductase activity. Following the switch from IR-HC to DR-HC therapy, there was a significant reduction in urinary cortisol and total GC metabolite excretion, which was most significant in the evening. There was an increase in 11β-HSD2 activity. Hepatic 11β-HSD1 activity was not significantly altered after switching to DR-HC, but there was a significant reduction in the expression and activity of 11β-HSD1 in subcutaneous adipose tissue.

**Conclusion:**

Using comprehensive in vivo techniques, we have demonstrated abnormalities in corticosteroid metabolism in patients with primary and secondary AI receiving IR-HC. This dysregulation of pre-receptor glucocorticoid metabolism results in enhanced glucocorticoid activation in adipose tissue, which was ameliorated by treatment with DR-HC.

Patients with adrenal insufficiency (AI) receiving essential glucocorticoid (GC) replacement therapy continue to have increased morbidity and premature mortality (1-4) with the exact mechanisms underpinning this increased mortality not fully elucidated. Patients with AI receiving GC replacement exhibit adverse metabolic and body composition profiles, including a propensity to central obesity and visceral fat disposition compared with control populations with similar body mass index (BMI) (5-8). Standard GC therapy with immediate release hydrocortisone (IR-HC) is unable to accurately replicate the physiological circadian cortisol rhythm, and together with the uncertainties of glucocorticoid dose adjustment and the absence of reliable biomarkers, patients receiving GC therapy may be over- or-underexposed to cortisol over a 24-hour period.

To mimic the physiological circadian rhythm of endogenous cortisol, novel modified-release hydrocortisone preparations have been developed. The dual-release formulation of hydrocortisone (DR-HC), Plenadren®, has been licensed for use in clinical practice and is taken once daily in the morning. In a prospective crossover study including patients with primary and secondary adrenal insufficiency, we have previously shown favorable metabolic changes following 12 weeks of DR-HC therapy with improvements in blood pressure, weight reduction, and improved quality of life (QoL) (9).

A further confounding issue in patients receiving exogenous GC therapy is the tissue-specific regulation of glucocorticoid action. Although circulating GC concentrations are regulated by the hypothalamic–pituitary–adrenal (HPA) axis, at the tissue level, GC action is also modulated through a series of enzymes, including the isozymes of 11β-hydroxysteroid dehydrogenases (11β-HSD). The 2 isozymes of 11β-HSD interconvert hormonally active cortisol (F) and inactive cortisone (E). 11β-HSD type 2 inactivates cortisol to cortisone in mineralocorticoid tissues such as the kidney, whereas 11β-HSD type 1 performs the reverse reaction converting inactive cortisone to active cortisol in key GC metabolic tissues including the liver and adipose tissue (10). Alteration in expression of these 11β-HSD isozymes in peripheral tissues modifies corticosteroid action. Tissues may thus be exposed to a relative excess of cortisol without any increase in cortisol secretion or circulating plasma cortisol concentrations. Glucocorticoids are also metabolized by the A-ring reductases, 5α-reductase (5αR), 5β-reductase, and 3α-hydroxysteroid dehydrogenase, to their tetrahydro metabolites. Thus, 5α-reduction contributes significantly to the metabolism and clearance of GCs (11). Several translational study techniques can be employed in clinical studies to investigate corticosteroid metabolism in vivo including urine steroid metabolite ratios in 24-hour collections (12), tissue-specific biopsies to measure gene expression, and dynamic tests such as cortisol generation profiles (13).

We hypothesize that corticosteroid metabolism is altered in patients with AI because of supraphysiological GC replacement therapy and the nonphysiological pattern of current IR-HC replacement therapy. The use of DR-HC, Plenadren®, may lead to improved markers of steroid metabolism because of the more physiological profile, which may restore normal GC metabolism. With this background, we aimed to perform a detailed prospective, crossover study to first characterize corticosteroid metabolism in patients with primary and secondary adrenal insufficiency and compared with an age-, gender-, and BMI-matched control population at baseline and after 12 weeks of DR-HC therapy.

## Methods

### Study Design and Population

We performed an investigator-initiated, open-labeled, multisite, prospective study at two University Hospitals in Ireland (Tallaght University Hospital and Beaumont Hospital). This study could not be blinded or placebo-controlled because of the risk of adrenal crisis in the study population with primary adrenal insufficiency (PAI) and secondary adrenal insufficiency (SAI). The inclusion and exclusion criteria of the study population have been previously published (9). Briefly, eligible patients were male and female patients aged ≥ 18 years, with a diagnosis of AI, either PAI or SAI, who were on immediate-release hydrocortisone replacement therapy, without any adjustment in hormone replacement for at least 3 months before study entry. Control participants were healthy individuals recruited via local advertisement and a diagnosis of adrenal insufficiency was excluded by clinical and biochemical parameters.

The study was approved by the Joint Research Ethics Committee of Tallaght University Hospital/St James's Hospital and the Beaumont Hospital Research Ethics Committee. Written informed consent was obtained from all patients before participation. All patients had an emergency kit and a steroid emergency card and received education regarding the management of an adrenal crisis.

This study was registered with ClinicalTrials.gov as NCT03282487.

### Clinical Protocol

**Figure 1.**
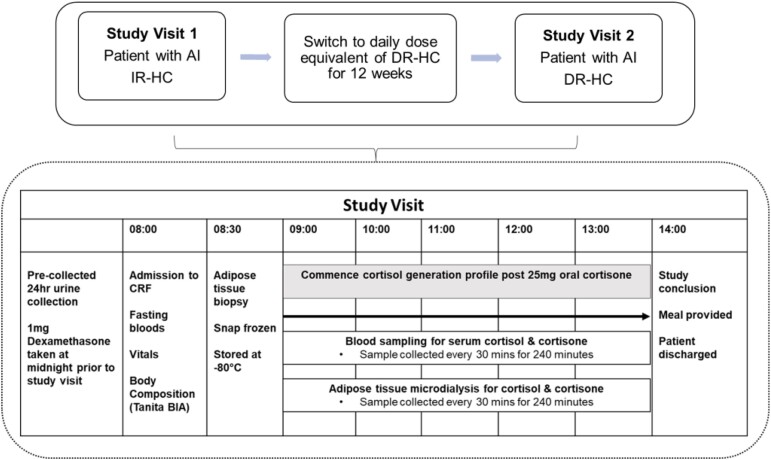
**Study protocol.** Study visit 1 completed on IR-HC and visit 2 completed after 12 weeks of DR-HC. Patients fasted for the duration of the cortisol generation curve. Healthy control participants did not undergo the adipose tissue biopsy. AI, adrenal insufficiency; BIA, bioelectric impedance (BIA); DR-HC, dual-release hydrocortisone; IR-HC, conventional immediate-release hydrocortisone.

After screening for eligibility and obtaining informed consent, study participants attended the research unit in the Department of Endocrinology, Tallaght University Hospital, or the Clinical Research Facility, Beaumont Hospital, after an 8-hour fast on 2 separate occasions, visit 1 and visit 2, for a day of integrated assessments (Fig. 1). On visit 1, patients presented for baseline evaluation on IR-HC therapy. Thereafter, if biochemical investigations were within the normal reference range, patients were switched from IR-HC to the daily dose equivalent of once-daily DR-HC, Plenadren®, per the summary of product characteristics, for 12 weeks. At the end of the intervention treatment period, patients presented for visit 2. Thereafter, patients were switched back to their usual IR-HC regimen because Plenadren was not available in clinical practice in Ireland at the time of the study. Patients were followed up in the outpatient clinic according to the standard surveillance protocol of the clinic. Control participants presented after an overnight fast on a single occasion and underwent the same biochemical investigations except for the adipose tissue biopsy and microdialysis.

At each visit (before and 12 weeks after DR-HC), patients presented at 8 Am, having taken 1 mg of dexamethasone at 11 Pm/midnight the night before the visit. A complete physical examination was performed in addition to baseline anthropometric assessment, which included body composition analysis with a bioimpedance body composition analyzer (Tanita BC418 MA for patients recruited in Tallaght University Hospital and Tanita DC360 S for patients in Beaumont Hospital). Baseline blood was drawn for fasting laboratory investigations including routine renal/bone/liver profiles, fasting total cholesterol, high-density lipoprotein- and low-density lipoprotein-cholesterol, triglycerides, hemoglobin A1c, C-reactive protein, and full blood count using in-hospital assays.

### Measurement of Corticosteroid Metabolism

#### Global corticosteroid metabolism

Immediate-release HC results in peaks and troughs in serum cortisol levels over the day (14), which may not be reflected in a 24-hour urine collection because this provides a global assessment of urinary steroid excretion. Therefore, all study participants collected 24-hour urine in 8-hourly intervals, in 3 separate containers (ie, bottle A, urine from 8.00 Am-4.00 Pm; bottle B, urine from 4.00 Pm-midnight; and bottle C, urine from midnight-8.00 Am). This was performed in the week before visit 1 (on IR-HC), and before visit 2 (12 weeks after DR-HC) but not in the 24 hours when dexamethasone was taken (ie, not the day before each visit) because this would interfere with steroid metabolite excretion. The total urine volume for each 8-hourly collection was recorded and two 20-mL volumes were preserved for storage at −80 °C until analysis for quantitative data on the urinary excretion of individual cortisol metabolites could be performed.

Urinary steroid metabolite excretion analysis was carried out by liquid chromatography-tandem mass spectrometry (LC-MS/MS) in the Institute of Metabolism and Systems Research, University of Birmingham, UK, using a Waters Xevo mass spectrometer with Acquity uPLC system, as previously described (15). The systemic relative 5αR activity was assessed by the ratio of 5α-tetrahydrocortisol (5α-THF)/tetrahydrocortisol (THF). The ratio of (THF + 5α-THF)/tetrahydrocortisone (THE) was used as a marker of 11β-HSD1 activity, and the ratio of urinary cortisol:urinary cortisone (urinary F/E) as a reflection of 11β-HSD2 activity. Total GC metabolite excretion was assessed as the sum of 5αTHF + THF + THE + cortols + cortolones + cortisol + cortisone. Patients' results were compared with an age-, gender-, and BMI-matched healthy control database established by the Institute of Metabolism and Systems Research.

#### Hepatic corticosteroid metabolism: cortisol generation curves

After baseline fasting bloods were taken, participants were given 25 mg of oral cortisone acetate. Following this, serial 30-minute serum samples were taken for 240 minutes. Measuring cortisol generated over time from oral cortisone acetate (in a dexamethasone-suppressed state) results in a curve representing 11β-HSD1 activity predominantly in the liver (16, 17). The serum samples stood at room temperature for 30 minutes to facilitate clotting before being centrifuged at 3000 rpm for 15 minutes, stored in 1-mL aliquots and at −80 **°**C until analysis. Serum cortisol and cortisone were analyzed by LC-MS/MS as previously described (18). For cortisol, analytical performance characteristics were as previously described (18). For cortisone, inter-assay imprecision was 5.5%, 3.9%, and 3.8% at concentrations of 5.0, 50.0, and 150 nmol/L, respectively. Mean recoveries ranged from −9% to 104% over concentrations of 63 to 500 nmol/L and ion suppression was found to be negligible (<10%). The limit of quantitation was 2.5 nmol/L, and the assay was free from analytical interferences.

#### Assessment of adipose tissue cortisol metabolism: adipose tissue microdialysis

Adipose microdialysis was carried out, as described by Tomlinson et al (16). After cleaning the skin with iodine solution, a CMA63® microdialysis catheter (CMA Microdialysis, Stockholm) was inserted into the subcutaneous adipose tissue, approximately 10 cm lateral and 5 cm inferior to the umbilicus. As per the serum cortisol generation curves, participants were given 25 mg of oral cortisone acetate (in a dexamethasone-suppressed state) and after a flush sequence (15μL over 5 minutes), microdialysis was performed at a rate of 0.3 μL per minute, with serial 30-minute microdialysis samples taken for 240 minutes. Microdialysis vials were stored at −80 °C until analysis. Each microdialysis sample was manually aspirated and prepared and analyzed on the Ultradian LCMS/MS Platform using a modified method as previously described (19). The assay precision for cortisone was 3.3% to 5.0% relative standard deviations (RSD), and the accuracy ranged from 94% to 104%. The assay precision for cortisol was 2.8% to 5.8% RSD, and the accuracy ranged from 98% to 100%. Lower limit of quantification was 69 pmol/L for both cortisol and cortisone. Because of low volumes of dialysate in some microvial samples, levels below 69 pmol/L have been reported but may have reduced accuracy and precision.

#### Assessment of genes related to adipose tissue GC action: adipose tissue biopsy

Subcutaneous adipose tissue biopsies were performed on the patient study population at baseline (on IR-HC) and the end of the 12-week treatment cycle with DR-HC. Subcutaneous adipose tissue from the abdominal wall was taken at the level of the umbilicus, approximately 4 to 7 cm lateral to the umbilicus. After local anaesthetic preparation of the area, a 14G biopsy needle was inserted and suction applied to the syringe by using a 4.5-mL vacutainer, to obtain a sufficient sample in the syringe. The collected sample was immediately snap-frozen in liquid nitrogen and stored at −80 °C until analysis.

Total RNA was extracted using the Tri-Reagent protocol. RNA concentration and purity were assessed using NanoDrop Spectrophotometer technology (ND1000, Thermo Scientific, Wilmington, DE, USA). One microgram of total RNA was used for reverse transcription and diluted to a final concentration of 50 ng/µL. Reverse transcription was carried out using TaqMan Reverse Transcription Reagents (Applied Biosystems Ltd). In this protocol, gRNA was reverse transcribed following the manufacturer's protocol. All reactions were incubated in a thermal cycler using the recommended cycling parameter: 25 °C for 10 minutes, 37 °C for 30 minutes, 95 °C for 5 minutes, and 4 °C indefinitely. The cDNA was stored at −20 °C until PCR performed. For quantitative PCR amplification of cDNA, all probes and primers were supplied by Applied Biosystems/Life Technologies as expression assays (TaqMan gene expression assays, catalog number 4331182:11βHSD1, Hs01547870_m1; 11βHSD2, Hs00388669_m1; NR3C1, Hs00353740_m1) and used following the manufacturer's protocol. All reactions were normalized against the housekeeping genes, 18S rRNA, glyceraldehyde-3-phosphate dehydrogenase, and peptidylprolyl isomerase A (20). Expression levels were determined using the ABI PRISM 7900HT Sequence Detection System (Thermo Fisher Scientific).

## Statistical Analysis

The normality of quantitative variables was tested with the Shapiro-Wilk test. The baseline characteristics of the groups were presented as mean (SD) or median (interquartile range [IQR]) as appropriate. The differences between the posttreatment and baseline data were evaluated with paired *t*-tests in a single group for quantitative variables and χ^2^ for categorical variables or the appropriate nonparametric test. Subgroup analysis was done to report the significance of treatment-by-subgroup interaction.

Real-time PCR data were obtained as Ct values (Ct = cycle number at which logarithmic PCR plots cross a calculated threshold line) and used to determine ΔCt values [ΔCt = (Ct of the target gen–) – (Ct of the reference gene)]. All statistical analysis of real-time PCR data was performed on mean ΔCt values between different treatment groups.

Significance was defined for *P* values less than 0.05. Statistical analysis was performed using GraphPad Prism version 8.2.0 for Windows, GraphPad Software, San Diego, California, USA.

## Results

The baseline characteristics of the study population and the impact of DR-HC on blood pressure, body composition, and QoL have been previously published (9). The study population comprised 21 patients with PAI and 30 patients with SAI who completed both visit 1 and visit 2 (after 12 weeks of DR-HC). There were more female patients in the PAI group (n = 12, 57%) and more males in the SAI group (n = 22, 73%). The study patients were on a median daily dose of 20 mg (IQR 15-20 mg) of IR-HC at study entry.

### Global Corticosteroid Metabolism: Urinary Steroid Excretion

#### Patients on IR-HC vs controls

**Figure 2.**
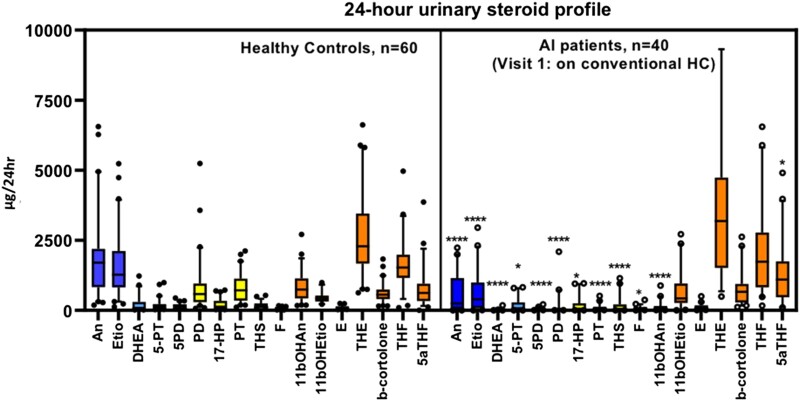
Steroid metabolite excretion (μg/24 hours) assessed by LC-MS/MS in healthy controls (n = 60) and patients with adrenal insufficiency receiving IR-HC (n = 40). The metabolites were divided into metabolites of androgens and precursors (blue), mineralocorticoids and precursors (green), glucocorticoid precursors (yellow), and glucocorticoids (orange). Box-and-whisker plots represent mean steroid excretion concentration and 5th and 95th percentiles. Significance = *P* value < .05 compared with healthy controls; *P < .05; **P < .01; ***P < .001; ****P < .0001.

We investigated urinary steroid excretion in a subcohort of our patient population and compared this with a healthy age-, gender-, and BMI-matched control population. A summary of patient and control characteristics with available 24-hour and diurnal (8-hourly interval) urinary steroid excretion is provided in Table 1. We observed significantly lower 24-hour excretion of androgen metabolites and all classes of steroid precursor metabolites; androgen, mineralocorticoid, and glucocorticoids, in patients with AI requiring GC replacement therapy compared with healthy controls (Fig. 2).

**Table 1.** Demographic data for patients with adrenal insufficiency receiving IR-HC (visit 1) who underwent 24-hour urinary steroid profiling and diurnal urinary steroid profiling, and available age- and BMI- matched control data

Characteristic	Patients with AI on IR-HC (visit 1)	Control cohort with 24-h urine collections	Control cohort with diurnal urine collections
Male/female (n/n)	20/20	30/30	10/8
Age (mean, SD) (y)	46 ± 12	48 ± 12	44.9 ± 11
AI phenotype			
Primary AI (n)	21	NA	NA
Secondary AI (n)	19		
HC dosage mg/d (median, IQR)	20 (16.25-20)	NA	NA
BMI kg/m^2^ (median, IQR)	26.6 (24.4-30.6)	26.9 (25.1-29)	27.0 (24-30)

Abbreviations: AI, adrenal insufficiency; BMI, body mass index; IQR, interquartile range; IR-HC, immediate release hydrocortisone.

**Figure 3.**
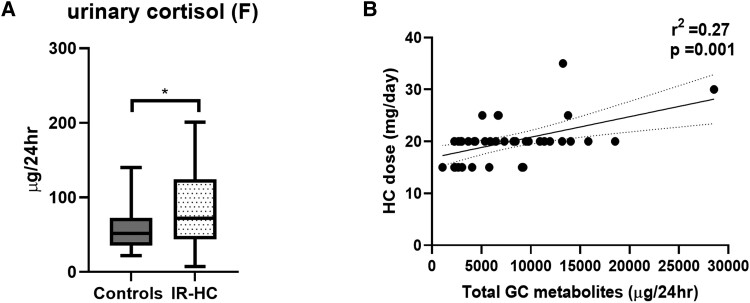
(A) 24-hour urinary excretion of cortisol in patients with adrenal insufficiency receiving IR-HC (n = 40) and healthy age- and BMI-matched control population (n = 60). (B) Correlation of 24-hour urinary excretion of total glucocorticoid (GC) metabolites with hydrocortisone dose in patients with AI. Box-and-whisker plots represent mean steroid excretion concentration and 5th and 95th percentiles. Significance = *P* value < .05.

Patients with AI receiving IR-HC (median, 20 mg [IQR, 16.25-20 mg]) had a higher median 24-hour excretion of urinary cortisol compared with healthy controls (72.1 µg/24 hour [IQR 43.6-124.2] vs 51.9 µg/24 hour [35.5-72.3], *P* = .02), Fig. 3A. Additionally, we observed a significant correlation between the hydrocortisone dose and total 24-hour urinary GC metabolite excretion in the patient population (*r*^2^ = 0.27, *P* < .001), Fig. 3B.

Within the diurnal urine excretion profiles, we observed a significant increase in the urinary excretion of the cortisol metabolite 5α-THF (median, 403.4 [IQR, 129.6-603.6] µg/8 hours vs 196.5 [IQR, 113.7-254.1] µg/8 hours, *P* = .04) in the morning urine collection, but there was no significant increase in total urinary cortisol (F) excretion or total GC excretion across the diurnal collections in patients receiving IR-HC compared with controls. We observed a significantly lower median urinary cortisol (F) excretion (3.4 [IQR, 1.5-13.0] µg/8 hours vs 23.1 [IQR, 3.3-33.6] µg/8 hours, *P* = .02) compared with healthy controls in the nighttime collection.

**Figure 4.**
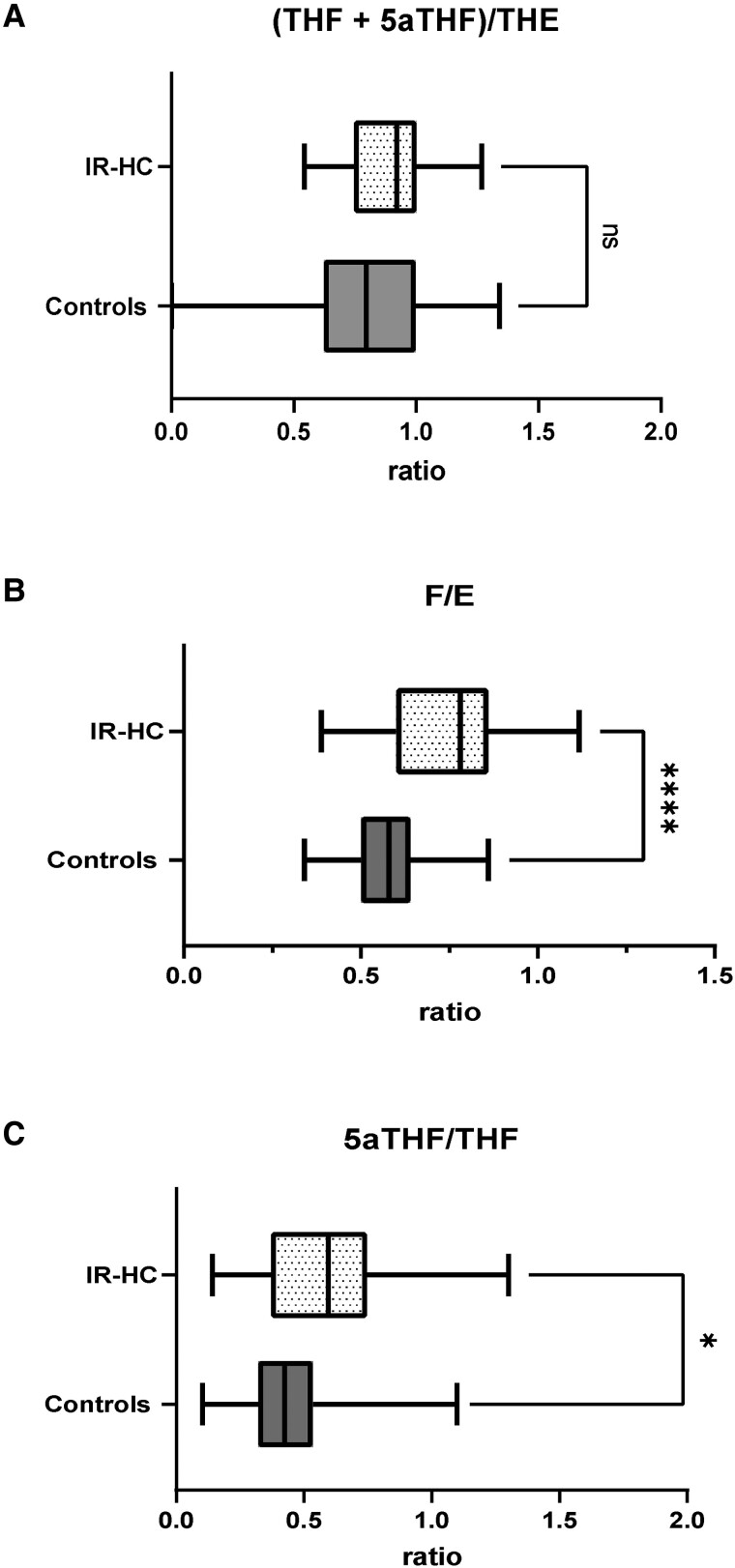
Twenty-four-hour urinary steroid excretion ratios in patients with adrenal insufficiency at baseline receiving IR-HC (n = 40) and healthy age- and BMI-matched controls (n = 60). (A) (THF + 5α-THF)/THE (measure of global 11β-HSD1), (B) urinary F/E (cortisol/cortisone) excretion (measure of 11β-HSD2), and (C) 5α-THF/THF (measure of 5α reductase activity). Box-and-whisker plots represent mean steroid excretion concentration and 5th and 95th percentiles. Significance = *P value < .05, **P value <.01.

When investigating the activity of key enzymes involved in steroid metabolism, we observed a significant increase in the ratio of 24-hour urinary cortisol/cortisone excretion in patients with AI compared with control population (ratio, 0.78 [IQR, 0.6-0.86] vs 0.58 [IQR, 0.5-0.64], *P* < .0001); however, there was no difference in the activity of 11β-HSD1 as indicated by lack of significant change in the ratio of THF + 5α-THF/THE. We also observed a significant increase in the ratio of 5α-THF/THF in the patient population (ratio 0.57 [IQR, 0.36-0.76] vs 0.43 [IQR, 0.32-0.54], *P* = .02), reflecting an increase in 5αR activity in patients receiving IR-HC therapy (Fig. 4).

#### Effect post-dual-release hydrocortisone

**Figure 5.**
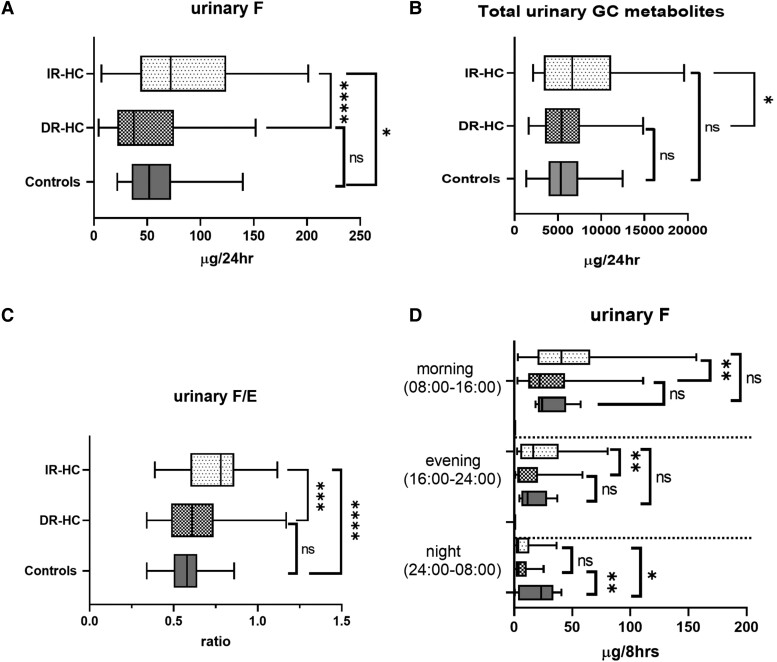
The 24-hour urinary excretion of (A) cortisol (F), (B) total glucocorticoid (GC) metabolites, (C) urinary cortisol/cortisone (F/E) ratio, and (D) diurnal 8-hour urinary excretion of cortisol, in patients with AI on IR-HC (visit 1), 12 weeks after DR-HC (visit 2), and healthy age- and BMI- matched controls. 5α-THF, 5 allo tetrahydrocortisol; DR-HC, dual-release hydrocortisone; E, cortisone; F, cortisol; GC, glucocorticoids; THE, tetrahydrocortisone. Box-and-whisker plots represent mean steroid excretion concentration and 5th and 95th percentiles. Significance = *P* value < .05; ns, not significant; *P < .05; **P < .01; ***P < .001.

Following the switch from IR-HC to DR-HC therapy, there was a significant reduction in urinary cortisol excretion compared to baseline (median, 72.1 [IQR, 43.6-124.2] μg/24 hours vs 37.4 [IQR, 22.2-75.3] μg/24 hours, *P* < .001), in addition to a reduction in total GC metabolite excretion (visit 1: median, 6659 [IQR, 3358-11151] μg/24 hours; visit 2: median, 5438 [IQR, 3515-7562] μg/24 hours, *P* = .015), Fig. 5A–B.

After 12 weeks of DR-HC therapy, patients had significantly lower urinary cortisol excretion in the morning (22.0 [IQR, 12.13-43.9] µg/8 hour vs 40.6 [IQR, 20.1-69.9] µg/8 hour, *P* = .002) and evening (4.3 [IQR, 2.6-20.4] µg/8 hour vs 16.4 [IQR, 5.4-38.2] µg/8 hour, *P* = .0016) compared with IR-HC therapy but not significantly lower than the healthy control population, Fig. 5D. There was no significant change in urinary cortisol excretion overnight after 12 weeks of DR-HC compared with baseline, however, urinary cortisol excretion was significantly lower than controls.

We observed a significant reduction in the urinary cortisol/cortisone ratio, indicating increased 11β-HSD2 activity, after 12 weeks of DR-HC compared with baseline (0.61 [IQR, 0.49-0.74] vs 0.78 [IQR, 0.6-0.86], *P* < .001), Fig. 5C. There was no significant difference in the global activity of 11β-HSD1 or global 5αR activity in patients after 12 weeks of DR-HC.

#### Hepatic corticosteroid metabolism (cortisol generation profile)

**Figure 6.**
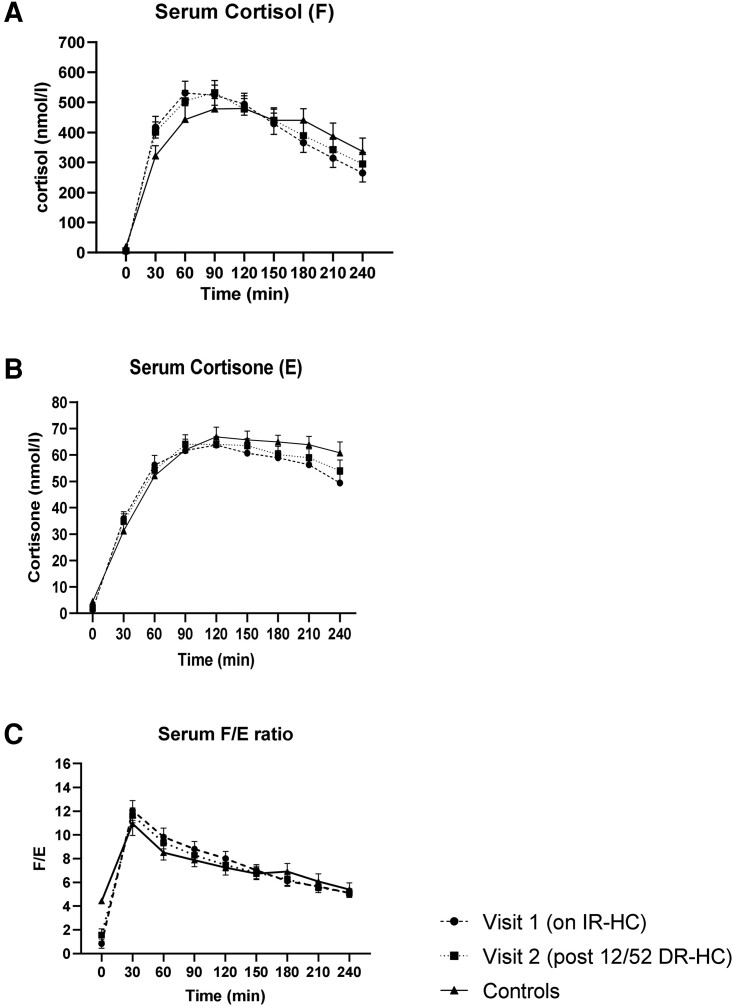
Cortisol generation time curves. Analysis of serum (A) cortisol, (B) cortisone, and (C) cortisol/cortisone (F/E) ratio after 25 mg of cortisone acetate, in patients with adrenal insufficiency receiving IR-HC (visit 1, n = 22) and after 12 weeks of DR-HC (visit 2, n = 22) and in healthy matched controls (n = 11). Data are expressed in mean (standard error of the mean).

Serum cortisol generation profiles (serum cortisol and cortisone after 1 mg of dexamethasone ingestion) measured via LC-MS/MS were available in 22 patients (11 males) on IR-HC (visit 1) and post 12 weeks of DR-HC (visit 2) and 11 controls (5 males), who were matched by age and BMI. Given the limited number of control data available for this analysis, we did not subdivide the cohort by gender because this would reduce the power of the analysis. All patients (n = 22) and healthy controls (n = 11) suppressed their morning fasting cortisol levels to <50 nmol/L following 1 mg dexamethasone ingestion the previous night. As expected (because of the preexisting AI), the mean fasting cortisol concentration level after dexamethasone suppression was significantly lower in patients with AI at baseline compared with the healthy control population (3.3 ± 7.4 vs 20.0 ± 4.9 nmol/L, *P* < .0001). Patients with AI on IR-HC reached a higher peak mean cortisol level following oral cortisone (531 ± 183 nmol/L vs 480 ± 138 nmol/L, *P* = .42) at an earlier timepoint of 60 minutes vs 120 minutes in the control population; however, this was not statistically significant, Fig. 6A. Interestingly, the healthy control patients had higher serum cortisone concentrations than patients with AI at 240 minutes after cortisone ingestion (49 ± 16 nmol/L vs 61 ± 14 nmol/L, *P* = .04), Fig. 6B.

After 12 weeks of DR-HC, there was no significant difference in the mean fasting cortisol concentrations after dexamethasone suppression (6.3 ± 9.6 nmol/L vs 3.3 ± 7.4 nmol/L, *P* = .15). The peak mean cortisol levels generated following cortisone acetate were similar at baseline and after DR-HC (531 ± 183 nmol/L vs 532 ± 189 nmol/L, *P* = .9). We assessed the ratio of serum cortisol to cortisone, a measure of hepatic 11β-HSD1 activity, in the patient population and the controls. There was no statistical difference in the ratio of serum cortisol/cortisone observed at any timepoint after 12 weeks of DR-HC, Fig. 6C.

### Adipose Tissue Corticosteroid Metabolism

#### Subcutaneous adipose tissue microdialysis

**Figure 7.**
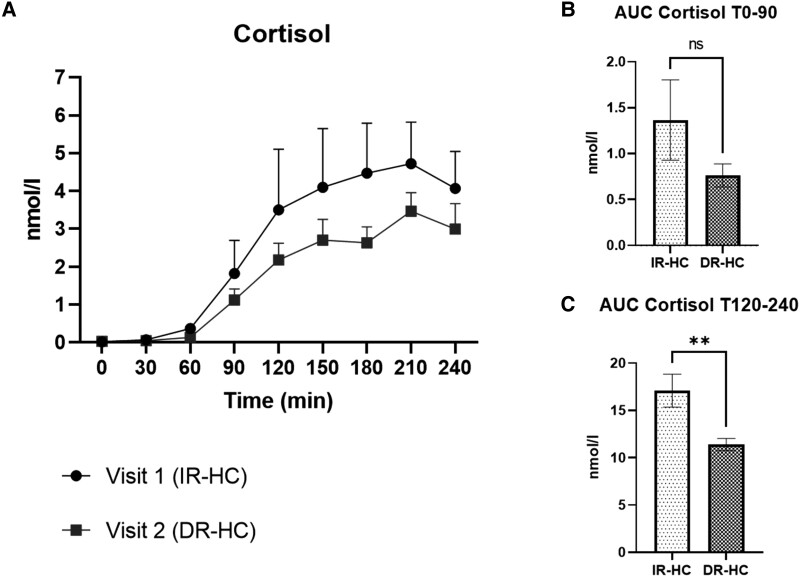
Microdialysis subcutaneous adipose tissue analysis in patients with AI receiving IR-HC (visit 1) and after 12 weeks of DR-HC (visit 2). (A) Microdialysis subcutaneous adipose tissue generation of cortisol after ingestion of 25 mg oral cortisone acetate. (B) Area under the curve (AUC) analysis of subcutaneous adipose tissue cortisol generation at time 0 to 90 minutes after cortisone acetate. (C) Time 120-240 minutes after cortisone acetate. Data are expressed in mean (standard error of the mean). Significance = *P* value < .05; ns, not significant; *P < .05; **P < .01; ***P < .001. No statistically significant difference between individual cortisol concentrations at each timepoint from visit 1 and visit 2 (A); however, there was a difference in AUC cortisol 120-240 minutes after ingestion of cortisone acetate (C).

Adipose tissue microdialysis samples were analyzed in 27 study patients with adrenal insufficiency (18 SAI, 9 PAI; 15 male,12 female). Cortisol generation profiles (measurement of cortisol and cortisone after 1 mg of dexamethasone ingestion) were measured via LC-MS/MS on IR-HC (visit 1) and after 12 weeks of DR-HC (visit 2). There was a reduction in cortisol concentrations in the subcutaneous adipose tissue dialysate after 12 weeks’ of DR-HC, with a significant reduction between timepoint 120 to 240 minutes after oral cortisone ingestion (17.1 ± 9.1 nmol/L vs 11.4 ± 3.0 nmol/L, *P* = .007), Fig. 7C.

#### Subcutaneous adipose tissue gene expression

**Figure 8.**
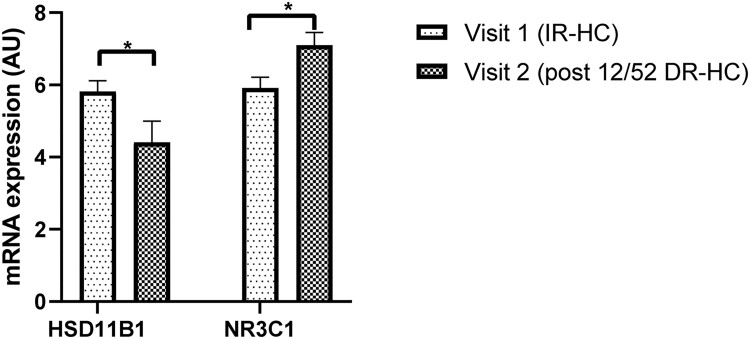
mRNA expression of HSD11B1 and glucocorticoid receptor (GR, NR3C1) from subcutaneous adipose tissue in patients receiving IR-HC (visit 1) and after 12 weeks of DR-HC (visit 2). Data expressed in arbitrary units (AUs) and as mean ΔCt ± standard error of the mean, significance = *P* < .05.

Subcutaneous adipose tissue biopsies were available for analysis in 10 patients before and after 12 weeks of DR-HC. We did not have subcutaneous adipose tissue available in the control population. Expression of 11β-HSD1 (HSD11B1) was significantly lower in subcutaneous adipose tissue after DR-HC compared with IR-HC (mean ΔCt before and after DR-HC, 5.8 ± 1.0 vs 4.4 ± 1.8, respectively, *P* = .03), whereas there was a significant increase in the expression of the glucocorticoid receptor (GR) gene, NRC31 (mean ΔCt before and after DR-HC, 5.9 ± 1.0 vs 7.1 ± 1.1, respectively, *P* = .02), Fig. 8.

## Discussion

This prospective study characterizes, for the first time, global, liver, and adipose tissue-specific corticosteroid metabolism in patients with primary and secondary adrenal insufficiency. We also report the differential impact of immediate-release hydrocortisone therapy and treatment with dual-release HC (Plenadren®) on steroid metabolism in AI.

There is a paucity of data investigating tissue corticosteroid metabolism in patients with AI receiving GC replacement therapy. In a cross-sectional study of patients with SAI, Sherlock et al demonstrated significant abnormalities in markers of corticosteroid metabolism in patients receiving IR-HC (21). A recently published study by Espiard and colleagues (22) focused on patients with primary adrenal insufficiency receiving DR-HC and observed significant alterations in the urinary steroid metabolome during DR-HC therapy compared with a 3-times-daily regimen of IR-HC. However, to our knowledge, no previous study has concurrently assessed both global and tissue-specific glucocorticoid metabolism as this study has done.

In our study, there was a significant difference in the 24-hour urinary steroid profile in patients with AI receiving IR-HC compared with healthy gender-, age-, and BMI-matched controls. We observed a significant difference in the 24-hour excretion of urinary cortisol in patients receiving IR-HC compared with controls, supporting data published in patients with SAI (21); however, there was no significant difference between total GC metabolite excretion between patients with AI and the control group. Espiard et al, using gas chromatography-MS measurement of 24-hour urinary steroids, reported significantly increased urinary GC metabolites compared with a healthy matched control population (22). This is in contrast to the findings in our study, which is potentially explained by the physiological daily dose of HC our study population received (median, 20 mg [IQR, 16.25-20]) compared with the study by Espiard et al where the study population received a clearly supraphysiologic mean daily HC dose of 30.1 mg (±5.5) (23). We did observe a significant positive correlation between HC dose and total urinary GC metabolite excretion in our patients, highlighting the importance of the daily HC dose in patients with AI with regard to overall tissue exposure to GC.

Following 12 weeks of dual-release hydrocortisone, we observed a significant reduction in 24-hour urinary cortisol and total GC metabolite excretion compared with patients receiving IR-HC; by the end of 12 weeks, this excretion rate was not different from healthy controls. A reduction in urinary cortisol after 12 weeks of DR-HC suggests reduced global corticosteroid exposure. This is likely to result in metabolic benefits, given the observation in the study by Sherlock et al that urinary cortisol metabolites correlate positively with central-to-thigh fat ratio, as assessed by dual energy X-ray absorptiometry in patients receiving HC (21). This reduction may account for the favorable changes in metabolic and QoL outcomes we observed in our study population (9).

Immediate-release HC results in peaks and troughs in serum cortisol over the day (14), which may not be reflected in a 24-hour urine collection because this provides a global assessment of urinary steroid excretion. Therefore, we assessed diurnal variation in urinary steroid excretion by urine collections performed in 8-hour intervals. In patients receiving DR-HC, there was a significant reduction in urinary cortisol in the morning and evening compared with IR-HC, to concentrations like those seen in the control group. Several studies have explored different HC regimes in patients with AI to identify the best strategy to replicate the distinct diurnal rhythm of cortisol secretion; however, those regimes inevitably result in steroid overreplacement or underreplacement across the 24-hour period (14, 24-27). Furthermore, even in healthy populations, high evening cortisol levels are associated with an increased risk of future glucose disturbance (28-30), a higher prevalence of vertebral fracture (31), and adverse effects on recognition memory (32). Plat et al showed that in a group of healthy males, administration of 50 mg of hydrocortisone at 5 Pm produced a more pronounced elevation in glucose levels and serum insulin and reduced insulin clearance, than when given at 5 Am (ie, near the peak of the normal circadian rhythm of the hypothalamic–pituitary–adrenal axis) (33). An elevation in nadir evening cortisol concentrations, as can occur in patients on thrice daily HC regimes, could be associated with disturbances in glucose tolerance (34). DR-HC therapy aims to avoid the peaks and troughs associated with immediate-release HC, potentially accounting for the favorable metabolic outcomes seen in our study patients and previous studies (9, 34).

11β-HSD isozymes modify the local action of glucocorticoids, whereas the A-ring reductases (5α- and 5β-reductase) inactivate cortisol (in conjunction with 3β-hydroxysteroid dehydrogenase) to its tetrahydro-metabolites (5α-THF and THE) (35). We observed a significant elevation in the ratio of 24-hour urinary cortisol/cortisone excretion in patients receiving immediate-release HC compared with controls, suggesting reduced 11β-HSD2 activity. After 12 weeks of DR-HC, there was a significant reduction in urinary cortisol/cortisone excretion, reflecting increased 11β-HSD2 activity, and thereby a reduction in tissue exposure to cortisol by inactivation to cortisone. Within the diurnal urine collections, we observed increased 11β-HSD2 activity (decreased urinary cortisol/cortisone ratio) across all collections, most significant in the evening collection (4:00 Pm-midnight) potentially offering a protective effect for the tissue, at a time at which physiological cortisol exposure is at its lowest (36).

The activity of the 11β-HSD2 in the kidney, protecting the mineralocorticoid receptor from inappropriate activation by cortisol, plays an important role in the maintenance of blood pressure control. The clinical importance of 11β-HSD2 is highlighted by congenital or acquired deficiencies in 11β-HSD2, which result in the syndrome of apparent mineralocorticoid excess presenting with classical features of hyperaldosteronism, including salt retention, potassium wasting, and hypertension (37-41). It has also been proposed that reduced activity of 11β-HSD2 could contribute to the pathogenesis of human essential hypertension, especially in its salt-sensitive form (42, 43). Previous clinical studies have shown increased urinary free cortisol/urinary free cortisone ratios, consistent with reduced 11β-HSD2 activity, in hypertensive patients (42, 44-46). The observation of decreased urinary cortisol/cortisone ratio in our study population may, therefore, in part explain the observed reduction in blood pressure after 12 weeks of DR-HC in our study population (9). The alteration in the urinary free cortisol/urinary free cortisone ratio may also reflect the reduction in substrate (cortisol) delivery to 11β-HSD2, which would result in a reduction in its activity; however, delineating these 2 hypotheses is beyond the scope of this study.

We also observed a significant increase in the ratio of 5α-THF/THF in the patient population at baseline, which infers an increase in 5αR activity. Studies have shown enhanced 5αR activity to be associated with obesity (47) and type 2 diabetes (48), with weight loss resulting in reduced 5αR activity and improvement in insulin sensitivity (49). Patients with polycystic ovary syndrome also exhibit alteration in 5αR, with increased 5αR activity correlating positively with markers of insulin resistance (50). Therefore, the observed alteration in cortisol A-ring reduction results in increased cortisol tissue exposure with potentially negative implications in the development of an adverse metabolic phenotype in patients receiving hydrocortisone replacement therapy.

The liver is the site of highest 11β-HSD1 expression, and immunohistochemistry studies have revealed that 11β-HSD1 expression in the human liver is localized centripetally with maximum expression around the central vein (13). Previous clinical studies have shown that after an oral dose of cortisone acetate, cortisol appears rapidly in the peripheral circulation (51, 52), in keeping with first-pass hepatic metabolism and localization of the oxoreductase isozyme, 11β-HSD1, to hepatocytes around the central vein (13). In our study, patients with AI receiving IR-HC had higher peak cortisol concentrations generated at an earlier timepoint compared with the control population after cortisone ingestion. This observation may reflect a difference in hepatic 11β-HSD1 activity between patients receiving chronic daily GC therapy and controls. However, we did not observe a significant difference in the ratio of serum cortisol/cortisone, reflective of hepatic 11β-HSD1 activity.

By contrast, we observed a reduction in the expression of 11β-HSD1 in subcutaneous adipose tissue after 12 weeks of DR-HC. Furthermore, we have shown using adipose tissue microdialysis that adipose tissue 11β-HSD1 activity was reduced after 12 weeks of DR-HC. Bujalska et al (53). first proposed that excessive activity of 11β-HSD1 within visceral adipose tissue could lead to increased adipose tissue concentrations of GCs and “Cushing disease of the omentum.” Subsequently, many studies have investigated whether 11β-HSD1 expression and activity in human adipose tissue are associated with obesity and insulin resistance (54-59). Most cross-sectional studies have suggested that adipose tissue 11β-HSD1 expression is increased in obesity and dysglycemia in parallel with decreased hepatic activity (17, 60). Furthermore, studies investigating the effects of endogenous GC excess or Cushing syndrome have shown that adipose-specific 11β-HSD1 knock-out mice were protected from hepatic steatosis and circulating fatty acid excess (10). Therefore, cortisol can increase expression of 11BHSD1 contributing to a “feed-forward loop” that might fuel adverse metabolic features (ie, cortisol increasing 11β-HSD1 expression generating more cortisol that further increases expression within the tissue). The reduction in 11β-HSD1 expression in subcutaneous adipose tissue, as seen in our study population, may potentially disrupt the feed-forward loop. Additionally, the observation of reduced urinary cortisol excretion after DR-HC may have an additional impact to lower 11BHSD1 expression, as observed in our adipose tissue biopsies.

We observed a significant increase in the expression of the NRC31 gene encoding the GR in subcutaneous adipose tissue after DR-HC. Cortisol regenerated by 11β-HSD1 binds to the GR within the cytoplasm and is then translocated to the nucleus, where it regulates the transcription of target genes, including those involved in inflammation (61). The GR has also been shown to transcriptionally activate 11β-HSD1, further amplifying GC action (62). Therefore, the observation of increased GR expression in adipose tissue in our patients after 12 weeks of DR-HC may be compensatory to the reduction in the expression and activity of 11β-HSD1. Several human studies have examined the association between adipose tissue GR mRNA levels and features of the metabolic syndrome and have shown no association (63) or, in fact, a negative correlation with the level of adiposity (55, 64, 65) and insulin resistance (55, 65, 66).

Our study is not without limitations. The study patients were not blinded to the treatment (for safety purposes). Our study population was switched from their IR-HC regime to the daily dose equivalent of DR-HC. Pharmacokinetic studies of DR-HC have shown that there may be an overall 24-hour reduction in cortisol exposure of approximately 20%, compared with an equivalent daily dose of thrice daily immediate-release hydrocortisone (23, 67). The lower GC exposure over 12 weeks could be a factor in altering expression of key enzymes in cortisol metabolism but these changes may also be related to the more physiological cortisol concentration throughout the day (see Fig. 5). Furthermore, our previous study reported improved QoL in the patient population with DR-HC; therefore, our study patients were not clinically hypoadrenal (9). It is difficult to decipher the relative contribution of weight loss and improved metabolic health on the outcomes reported in this study because it is well-recognized that weight loss does have an impact on both hepatic and adipose tissue cortisol metabolism and 11β-HSD1 activity (17, 49). However, this is the first study to comprehensively assess the effect of DR-HC on tissue cortisol metabolism (in multiple tissues), and this potential confounder needs to be further elucidated in future studies. Because of the intensive nature of the in vivo study protocol, a healthy control population was challenging to recruit; therefore, the size and clinical characteristics of the control groups for each experiment did differ. However a primary focus of the study was to assess the effect of DR-HC in the individual patient with the same genetic and physiological profile (ie, the patients themselves acting as a control group). Finally, we did not address racial differences in cortisol metabolism within our dataset as our study population were all Irish Caucasian.

In conclusion, this study demonstrates abnormalities in corticosteroid metabolism in patients with primary and secondary adrenal insufficiency receiving IR-HC, resulting in increased adipose tissue GC exposure. This dysregulation is ameliorated after 12 weeks of DR-HC treatment. These alternations represent a potential mechanism underlying beneficial metabolic effects of more physiologic GC replacement regimens.

## Data Availability

Some or all datasets generated during and/or analyzed during the current study are not publicly available but are available from the corresponding author on reasonable request.

## Abbreviations

5αR: 5α-reductase
5α-THF: 5 allo tetrahydrocortisol
11β-HSD: 11β-hydroxysteroid dehydrogenase
AI: adrenal insufficiency
BMI: body mass index
DR-HC: dual-release hydrocortisone
GC: glucocorticoid
GR: glucocorticoid receptor
IQR: interquartile range
IR-HC: immediate release hydrocortisone
LC-MS/MS: liquid chromatography-tandem mass spectrometry
PAI: primary adrenal insufficiency
QoL: quality of life
SAI: secondary adrenal insufficiency
